# Pemphigoïde bulleuse et maladie de Parkinson: à propos d'un cas

**DOI:** 10.11604/pamj.2017.28.111.12925

**Published:** 2017-10-04

**Authors:** Hasnaa Zaouri, Baderdine Hassam

**Affiliations:** 1 Service de Dermatologie, Centre Hospitalier Universitaire Avicenne, Faculté de Médecine et de Pharmacie Mohamed V, Rabat, Maroc

**Keywords:** Bullous pemphigoid, Parkinson´s disease, neurological diseases, Bullous pemphigoid, Parkinson's disease, neurological diseases

## Abstract

Bullous pemphigoid (BP) is an organ-specific autoimmune disease which can be associated with many pathologies including degenerative neurological diseases such as Parkinson's and Alzheimer's disease. Studies have suggested the possibility of cross-immune responses through “epitope spreading”. Bullous dermatosis has been reported to be secondary to neurological disease, at intervals ranging from a few months to a maximum of a few years. Then is it an unusual association or a causation? It has been suggested that drug consumption, decubitus lesions, traumatic events as well as reduced immunity can be triggers for BP in patients with neurological disease. We report the case of a 93-year old patient with a 10-year history of advanced Parkinson's disease, hospitalized for the treatment of a common bullous pemphigoid confirmed by histology and immunohistochemistry. The patient was treated with oral corticosteroids. After a week of treatment, the patient died due to septic shock. Neurological disorders represent a real risk factor for BP. BP could be considered as a marker for neurological disorder. These associations are of broad interest, because they can play a role in the etiopathogenesis of BP and contribute to a complete understanding of the causes of these neurodegenerative diseases.

## Image en médecine

La pemphigoïde bulleuse (PB) est une maladie auto-immune spécifique d'organe, qui peut être associée à de nombreuses pathologies, notamment à des maladies neurologiques dégénératives, comme la maladie de Parkinson et la maladie d'Alzheimer. Des études ont suggéré la possibilité de réactions immunitaires croisées selon le phénomène «epitope spreading». La survenue de dermatose bulleuse a toujours suivi l'apparition de la maladie neurologique à des intervalles allant de quelques mois à un maximum de quelques années. Alors s'agitil d'une association occasionnelle ou de causalité? Il a été suggéré que la consommation de médicaments, les lésions de décubitus, les événements traumatiques et le vieillissement de l'immunité peuvent être des facteurs déclenchants de BP au cours de maladies neurologiques. Nous rapportons le cas d'un patient de 93 ans, ayant comme antécédent une maladie de parkinson stade avancé depuis 10 ans, qui a été hospitalisé pour la prise en charge d'une pemphigoïde bulleuse typique, confirmé à l'histologie et l'immunohistochimie. Le patient a été traité par corticoïdes oraux. Après une semaine de traitement, le patient est décédé dans un tableau de choc septique. Les pathologies neurologiques constituent un véritable facteur de risque de survenue de PB. Cette dernière pourrait être considéré comme marqueur de désordre neurologique. Ces associations sont d'un intérêt majeur, car ils peuvent jouer un rôle dans l'étiopathogénie de la PB et peuvent participer à la compréhension de la base pour ces maladies neurodégénératives.

**Figure 1:**
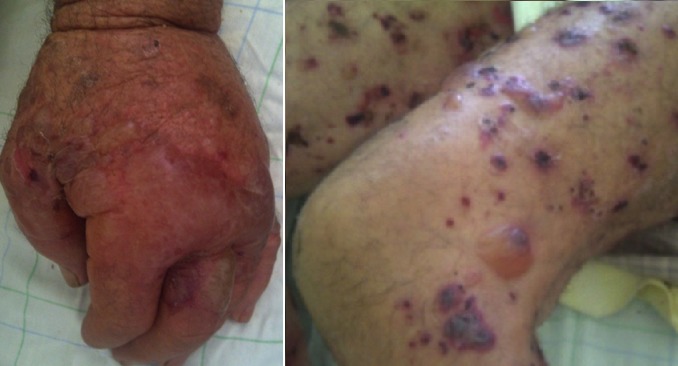
Bulles tendues à contenu clair et hémorragique

